# Functional Genomic Identification of Cadmium Resistance Genes from a High GC Clone Library by Coupling the Sanger and PacBio Sequencing Strategies

**DOI:** 10.3390/genes11010007

**Published:** 2019-12-20

**Authors:** Jinghao Chen, Chao Xing, Xin Zheng, Xiaofang Li

**Affiliations:** 1 Hebei Key Laboratory of Soil Ecology, Key Laboratory for Agricultural Water Resource, Center for Agricultural Resources Research, Institute of Genetics and Developmental Biology, Chinese Academy of Sciences, Shijiazhuang 050021, China; chenjinghao17@mails.ucas.ac.cn (J.C.); xingchao17@mails.ucas.ac.cn (C.X.); zhengxin@sjziam.ac.cn (X.Z.); 2 University of Chinese Academy of Sciences, Beijing 100049, China

**Keywords:** high GC DNA library, functional genomics, PacBio Sequel, *Cellulomonas*

## Abstract

Functional (meta) genomics allows the high-throughput identification of functional genes in a premise-free way. However, it is still difficult to perform Sanger sequencing for high GC DNA templates, which hinders the functional genomic exploration of a high GC genomic library. Here, we developed a procedure to resolve this problem by coupling the Sanger and PacBio sequencing strategies. Identification of cadmium (Cd) resistance genes from a small-insert high GC genomic library was performed to test the procedure. The library was generated from a high GC (75.35%) bacterial genome. Nineteen clones that conferred Cd resistance to *Escherichia coli* subject to Sanger sequencing directly. The positive clones were in parallel subject to in vivo amplification in host cells, from which recombinant plasmids were extracted and linearized by selected restriction endonucleases. PacBio sequencing was performed to obtain the full-length sequences. As the identities, partial sequences from Sanger sequencing were aligned to the full-length sequences from PacBio sequencing, which led to the identification of seven unique full-length sequences. The unique sequences were further aligned to the full genome sequence of the source strain. Functional screening showed that the identified positive clones were all able to improve Cd resistance of the host cells. The functional genomic procedure developed here couples the Sanger and PacBio sequencing methods and overcomes the difficulties in PCR approaches for high GC DNA. The procedure can be a promising option for the high-throughput sequencing of functional genomic libraries, and realize a cost-effective and time-efficient identification of the positive clones, particularly for high GC genetic materials.

## 1. Introduction

Base composition substantially impacts genome stability and evolution [1], and high-GC content is thought to be associated with high selective pressure [2]. It has been found that prokaryotic extremophiles particularly metal-resistant strains commonly possess high-GC genomes (Supplementary Table S1). For example, the 63 genomes currently found in GenBank of Cu resistant *Cupriavidus* strains have an average GC content of 66.2%, the 24 genomes of multi-metal resistant *Thiobacillus* strains have an average GC content of 62.6%, and the 45 genomes of radiation and/or metal tolerant *Deinococcus* strains have an average GC content of 67.3%. Zn-resistant *Comamonas* spp. have a GC content of 61.3–61.5% [3,4,5], and cadmium (Cd)-resistant *Mesorhizobium metalliduran* [6] and *Gluconacetobacter diazotrophicus* [7] have a GC content of 62.5% and 66.3%, respectively. Metagenomes from metal-rich mine tailings are also found to have a GC content of > 60%. For example, Mt Isa and Dexing copper tailings isolates were between 49% to 72% GC [8]. Therefore, functional characterization of prokaryotic genetic determinants for metal resistance will inevitably involve dealing with the high GC genomic materials.

Library-based functional genomics provides a premise-free exploration of novel functional genes [9,10]. Conventionally, PCR-based Sanger sequencing is applied to the candidate genes from functional screening [11]. However functional genomic exploration of high GC genetic material is difficult by such strategies. It is well known that the skew of GC content in PCR template sequences is associated with possible failures in PCR amplification [12], and thus Sanger sequencing of high GC genes is costly and time-consuming.

The next generation sequencing by Illumina is able to realize the high-throughput sequencing of high GC materials, yet is impractical for the pooled libraries at a reasonable cost and time-scale, because the sequencing-by-synthesis strategy used by Illumina has a potential for GC content bias during PCR amplification of templates [13]. The third generation sequencing platforms, typically by PacBio, provide a high-throughput mean to sequence environmental DNA [14]. Relative to the second generation sequencing technology, the PacBio sequencing strategy overcomes the difficulties in PCR bias for high GC DNA molecules [15]. Unfortunately, unlike the Illumina platforms, the PacBio sequencing technology is not able to sequence multiple libraries in parallel due to the lack of proper barcoding [16].

Here, we report a procedure coupling the Sanger and PacBio sequencing systems, which realizes the functional genomic identification of high GC target genes in a high-throughput fashion. PacBio single molecule real-time (SMRT) sequencing can provide a PCR independent and efficient way to generate long reads with uniform coverage and high consensus accuracy via recognizing the fluorescent signal on single phospholinked nucleotides [17]. This procedure was applied to a small-insert genomic DNA library of high GC content for the identification of Cd resistant genes. This procedure overcomes the difficulties in PCR approaches for high GC gene templates and realizes a cost-effective and time-efficient identification of the positive clones.

## 2. Materials and Methods

### 2.1. Experimental Design

**Figure 1:**
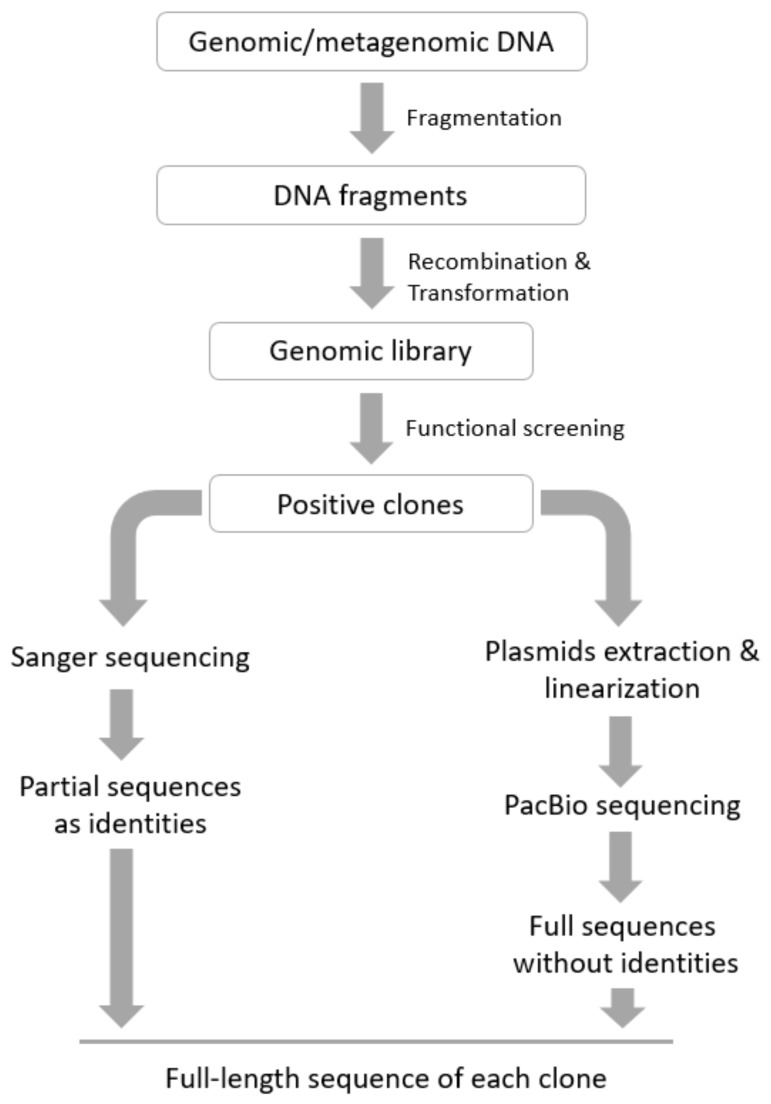
Workflow of the functional genomic procedure for a high (G + C)% library described in this study.

We designed a procedure (Figure 1) for the high-throughput sequencing of a high GC library constructed from the genome of a Cd tolerant strain, which couples the Sanger and PacBio sequencing approaches. Briefly, a genomic library is constructed through conventional procedures. After functional screening, the positive clones are subject to Sanger sequencing, and aliquots of them are in parallel subject to in vivo amplification in host cells. Then, the recombinant plasmids are extracted, linearized by dedicated restriction endonucleases (REs), and pooled together for PacBio sequencing. Finally, the partial sequences (as the identity of each sequence) from Sanger sequencing are aligned to the full-length sequences from PacBio sequencing to obtain the full-length sequence of each positive clone.

### 2.2. The Strain and Culture Conditions

The bacterial strain used for functional screening in this study was *E. coli* DH5α cultured in the Luria-Bertani (LB) medium at 37 °C. Ampicillin (100 mg L^−1^) was added to maintain the pUC118 plasmid and recombinant plasmids. A Cd resistant strain, namely the *Cellulomonas* sp. strain Y8, was isolated from a soil. Its physiological feature will be reported elsewhere.

### 2.3. DNA Extraction

Genomic DNA was extracted by using a cetyltrimethyl-ammonium-bromide (CTAB) method [18] with modifications. The cells were harvested by centrifugation at 4000× *g*, and disrupted by bead-beating for 10 min to break the cell wall. Then, a buffer solution (100 mmol L^−1^ Tris base, 100 mmol L^−1^ EDTA, 1.5 mol L^−1^ NaCl, 2% CTAB, 2% SDS) was added and samples were incubated at 65 °C for 30 min, followed by centrifugation at 10,000× *g* to collect supernatant. Finally, DNA was precipitated by using isopropanol and purified by using a DNeasy PowerClean Pro cleanup kit (QIAGEN, Hilden, Germany) according to the manufacturer’s protocols.

### 2.4. Full Genome Sequencing

To verify the derivation of the sequences obtained from the functional genomics procedure, the complete genome of *Cellulomonas* sp. strain Y8 was sequenced by using the Illumina HiSeq (Illumina, San Diego, CA, USA) and PacBio RS II platforms (Pacific Biosciences, Menlo Park, CA, USA) according to standard protocols. For HiSeq sequencing, genomic DNA was randomly fragmented and treated with an end prep enzyme mix. The size selection of adaptor-ligated DNA was performed, and then fragments of ca. 470 bp (with the approximate insert size of 350 bp) were recovered. Each sample was then amplified by PCR for eight cycles using P5 and P7 primers. The PCR products were cleaned up and validated using an Agilent 2100 Bioanalyzer (Agilent Technologies, Palo Alto, CA, USA), and quantified on a Qubit 3.0 Fluorometer (Invitrogen, Carlsbad, CA, USA). Then, libraries with different indices were multiplexed and loaded on an Illumina HiSeq instrument according to the manufacturer’s instructions (Illumina, San Diego, CA, USA). Cutadapt (v1.9.1) [19] was employed for quality control, and reads whose base groups with quality value below 20 at both ends, sequences containing more than 10% N base, or less than 75 bp in length were removed. The Illumina data was not assembled but used for correction.

For PacBio sequencing, genomic DNA was sheared, and then 10 Kb double-stranded DNA fragments were selected. DNA fragments were end-repaired and ligated with universal hairpin adapters. The library was sequenced in PacBio RS II instrument. PacBio reads were assembled using HGAP4/Falcon of WGS-Assembler 0.3 [20,21]. The genome was re-corrected with Pilon using the obtained Illumina data.

### 2.5. Functional Genomic Screening

In this study, we aimed at the Cd resistance related genes, most of which are <3000 bp. In addition, a longer insert of high GC may have difficulties in expression in the host *E. coli*. Therefore, a small-insert genomic DNA library was prepared to screen the positive clones with Cd resistance. Briefly, genomic DNA was partially digested with *Sau*3AI (New England Biolabs, USA) to produce DNA fragments, which were subject to electrophoreses on an agarose gel to harvest DNA fragments ranging from 1 to 3000 bp. The DNA fragments (150–200 ng) and linearized pUC118 vector (100 ng, with *Bam*H1 cohesive end, Takara Bio Inc., Japan) were mixed with T4 DNA ligase (New England Biolabs, USA) and incubated overnight at 16 °C according to the manufacturer’s protocols. A total of 10 μL ligation solution was subsequently transformed into 100 μL of commercial competent *E. coli* cells using a CaCl_2_-based chemical transformation method, followed by activation in 1 mL super optimal broth with catabolite repression (SOC) liquid medium for 60 min.

Cd resistance clones were screened out on solid LB medium plus 100 μg mL^−1^ ampicillin and 1 mmol L^−1^ Cd. In short, 15 μL of transformed cells were spread on the LB plate and cultured at 28 °C for 36 h. The strain harboring empty pUC118 was used as negative control. The colonies that were able to grow after 36 h cultivation were selected. The recombinant vectors were extracted from selected colonies and re-transformed into *E. coli* cells and screened to find those which could grow under a 1 mmol L^−1^ Cd stress. These were considered as positive clones harboring potential cadmium resistant genes. The capacity of library was calculated as described in our previous study [22] and the final genomic DNA library consisted of 2.2 × 10^5^ recombinant clones with an estimated capacity of 200 Mb.

### 2.6. Sanger and PacBio Sequencing of Amplicons

Both Sanger sequencing and PacBio sequencing were performed by GENEWIZ (Suzhou, China). For Sanger sequencing, using M13F and M13R primers with standard high GC template-focused optimizations. For PacBio sequencing, all recombinant vectors harboring target genomic DNA fragments were firstly amplified in vivo and extracted for the linearization by Restriction endonucleases (REs) in vitro. Different REs (*Bam*HI, *Hin*dIII, and *Xba*I, New England Biolabs, USA) were tested and those that could generate a single cleavage were used for the linearization. All linearized plasmids were then mixed together as a composite library. SMRTbell™ templates preparation was done using a DNA template prep kit. The binding of SMRT bell templates to polymerases was conducted using the DNA/Polymerase binding kit and sequencing primers v3 according to the manufacturer’s instruction. The prepared SMRTbell™ templates was bound with Magbead and loaded on a SMRT cell of PacBio Sequel platform and sequenced in PacBio SMRT instrument. PacBio SmartLink was then employed to address subreads and generate CCS (Circular consensus sequencing) sequences. This CCS sequence was then subject to comparison analysis with Sanger results. Both the forward and reverse partial reads of the Sanger results were mapped onto the CCS sequences to obtain the full-length tagged sequences.

### 2.7. Open Reading Frame Prediction and Annotation

ORF finder (https://www.ncbi.nlm.nih.gov/orffinder/) was employed to predict ORFs on all obtained sequences. The ORFs longer than 150 bp were translated into protein sequences, which were further characterized by BLAST search against the UniProtKB database (http://www.uniprot.org/blast/) to further match the optimal protein sequence and perform functional prediction (E-value less than 1 × 10^−30^, identity > 30%).

### 2.8. Drop Assay

To determine the extent of Cd resistance conferred by the clones, the re-transformed strains were cultured, harvested, and resuspended in water to a final OD_600_ of 1.0. The resuspended cells were then serially diluted, and a total of 5 μL of each dilution was inoculated onto the LB plates containing 100 μg mL^−1^ ampicillin and 1 mmol L^−1^ Cd. Strains harboring empty pUC118 were used as a negative control. The plates were incubated at 28 °C for 48 h and growth of the colonies was subsequently observed.

## 3. Results and Discussion

The strain used in this study, *Cellulomonas* sp. strain Y8, was able to survive on an LB plate with 8 mM Cd. The full genome of the strain was sequenced by PacBio SMRT sequencing. The Illumina sequencing resulted in 20,382,470 of clean reads with an average length of 148 bp, which were used for verifying the sequencing results of the clones. The complete genome comprised 4,475,991 bp of sequence with a 75.35% GC content (genome accession number CP041203) [23]. For the PacBio sequencing, it generated 232,404 sequences with an average length of 3620 bp and an N50 of 4551 bp. A small-insert genomic DNA library of *Cellulomonas* sp. strain Y8 was prepared for Cd stress screening as described in our previous study [22]. Finally, 19 Cd resistant colonies were detected, and the recombinant vectors were re-transformed into *E. coli* cells to verify their resistance.

In our pilot experiment, conventional PCR was applied to detect the inserted DNA fragments in the library, and as expected because of the extremely high GC content, no clear PCR products were observed. In Sanger sequencing, PCR failed mostly with early termination or attenuation (Table 1) even with high GC template-focused optimizations including modification of cycling conditions, use of modified nucleotides [24] and some additives such as dimethylsulfoxide [25], betaine [26], formamide, and bovine serum albumin [27]. Previous studies have shown that these additives increase the specificity of amplification products, but are far less effective than required at a reasonable cost [28]. When the template length (e.g., > 3000 bp) and number (> 100) increase, it is not feasible to perform Sanger sequencing for high GC genomic libraries.

**Figure 2:**
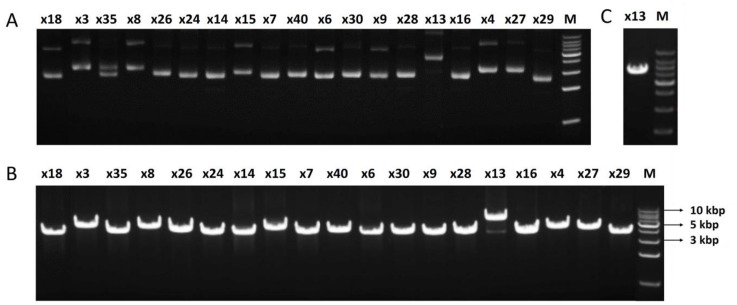
Screening of restriction endonucleases for the linearization of plasmids carrying the high GC amplicons. (**A**) Agarose gel (1% *w*/*v*) electrophoresis of the plasmids. Lanes: x18 to x29, plasmid x18 to x29; M, molecular weight ladder (1 kbp, Boehringer). (**B**) *Hin*dIII digestion of all plasmids, analyzed by gel electrophoresis. Lanes: M, molecular weight ladder (1 kbp, Boehringer); x18 to x29, plasmid x18 to x29. (**C**) *Xba*I digestion of x13. Lanes: x13, plasmid x13; M, molecular weight ladder (1 kbp, Boehringer).

To satisfy the requirements of PacBio sequencing [16] and final identification of the target clones, the plasmids carrying the target clones must be linearized to a single DNA fragment. Dedicated REs were selected based on known enzyme cutting sites and then tested in vitro (Figure 2). *Eco*RI and *Hin* dIII REs were firstly tested and *Hin*dIII was able to realize a single cleavage for all clones except the clone x13 (Figure 2B). *Xba*I was found to be suitable for the clone x13 (Figure 2C). It is worth noting that the REs used for linearization were dependent on the plasmid used for cloning, and in this study, the combination of *Hin*dIII and *Xba*I successfully satisfied the requirements.

For PacBio sequencing of the library, 117,538 of CCS sequences were obtained. After removal of pUC118 plasmid sequences, bulk full-length sequences of the target genes were obtained without identity. These sequences were further aligned with the partial sequences by Sanger sequencing (Table 1). The length of valid reads with acceptable sequencing quality ranged from 57–981 bp. This length is sufficient for a high-quality alignment with the PacBio sequences [29]. Some exceptions were excluded from the alignment including the clones x3, x4, x8, x18, x27 and x40 that was too short. As a procedure for practical use, the partial sequencing by the Sanger method can be minimized to around 20 bp if the sequencing quality is sufficient.

The identified sequences were further searched against the full genome of the strain Y8, to check whether there were artifacts or contaminating sequences. The results showed that all sequences were matched to a particular locus of the Y8 chromosome, with an identity of 94.32–99.53% (Table 1). These results indicate that the procedure developed here is successful for the sequencing of high GC clones from a functional genomic library in a time-efficient way. The mismatches between the identified clone sequences and the full genomic regions also indicated apparent sequencing errors of the amplicons from the PacBio platform, which is reported to be between 11–15% [17].

**Figure 3:**
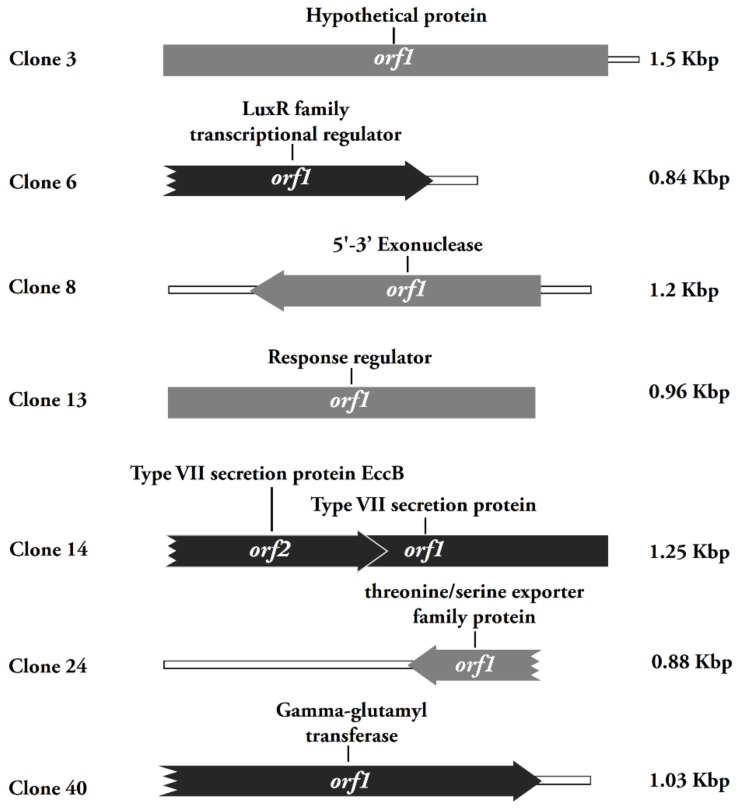
Schematic organization of the open reading frames identified in the Cadmium-resistance plasmids. Arrows indicate the directions of transcription of each ORFs in the plasmids. Left-facing ORFs are denoted by dark grey arrows; right facing ORFs are black. Truncated ORFs are indicated as incomplete arrows.

**Figure 4:**
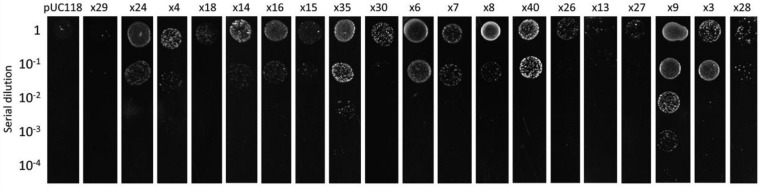
Cadmium resistance test of the candidate Cd-resistant clones identified in this study. Lane: pUC118, *E. coli* strain DH5α carried an empty vector pUC118 as control; x29 to x28, strain DH5α carried vector x29 to x28, respectively. Row: 1 to 10^−4^, the initial OD 600 nm value of the inoculated strain.

The genetic determinants positive in the Cd resistance test were then annotated. (Figure 3). These positive clones contained a LuxR family transcriptional regulator gene, a 5′–3′ exonuclease gene, a response regulator gene, two Type VII secretion genes, a threonine/serine exporter family gene, a γ -glutamyltransferase gene, and a hypothetical gene. Previous studies found that the Lux protein family was associated with metal resistance [30], however, the function of the LuxR family transcriptional regulator is still unclear. The clones x3, x4, and x18 contain a unique ORF that improved Cd resistance of *E. coli*. This ORF encodes a protein which is similar to a polymerase/histidinol phosphatase-like hypothetical protein from *Cyclobacterium qasimii* [31]. The γ -glutamyltransferase gene *ggt* carried by x40 encodes a glutathione (GSH) metabolism related protein/enzyme, whose function has been well studied in *E. coli* strain K12 [32]. It is worthy to note that GSH has been reported to play a role in Cd tolerance in a variety of cells [33,34,35,36,37]. These positive clones were further re-transferred into *E. coli*, and functional screening showed that most of these clones improved the growth of the hosts relative to the control (Figure 4). Among them, clone x9 showed the strongest resistance to Cd, and was able to survive with a rather low initial inoculum concentration (10^−4^). This suggests that these clones obtained from in vivo amplification are biologically functioning and convey Cd resistance to *E. coli*.

Overall, the current study developed a functional genomic procedure for identification of metal resistance genes from a high GC genomic library, by coupling the Sanger and PacBio sequencing platforms. We identified seven unique sequences from 19 clones that conveyed Cd resistance, which can be candidate genetic materials for developing bioremediation tools. The Illumina platform is powerful and able to sequence high-GC genomes, but the depth, quality, and cost is basically difficult to obtain a full genome [15]. The difficulties facing Illumina for high GC genomes/long DNA fragments are also why we developed the procedure here. The length of the clones identified in this study is however not very long, our experimental design can facilitate sequencing of genes as large as 3000 bp.

Considering that the sequencing cost increases drastically when the length of genomic targets increases for Sanger sequencing, and the cost of PacBio sequencing has been constantly falling, the procedure developed here can be a promising alternative option for the high-throughput sequencing of functional genomic libraries, particularly for high GC genetic materials.

## Figures and Tables

**Table 1:** Statistical information of the Sanger and PacBio sequencing.

Clone ID	Partial Length by Sanger Sequencing (Bp) (Forward, Reverse)	Full Length by PacBio Sequencing (Bp)	Genome Locus	GC Content (%)	Identity (%)
3,4,18	15, 604	1487	3835167–3836672	79.73	95.32
6,7,9,15, 16,29,30,35	100, 981	841	4380530–4381372	77.65	97.34
8,27	314, 57	1447	1213476–1214931	79.22	96.72
13,28	51, 618	930	1608787–1609730	78.16	94.32
14	572, 933	1258	2627868–2629126	81.09	99.53
24	7, 746	900	1780961–1781871	77.77	97.72
40	1, 93	1017	1342804–1343830	79.01	95.85

## Supplementary Materials

The following are available online at https://www.mdpi.com/2073-4425/11/1/7/s1. Table S1: Reported metal resistance prokaryotes and their genome GC content.
