# Supplementary material for: Functional Genomic Identification of Cadmium Resistance Genes from a High GC Clone Library by Coupling the Sanger and PacBio Sequencing Strategies
Source: Genes (Basel). 2019 Dec 20;11(1):7. doi: 10.3390/genes11010007 (PMC7016576; doi:10.3390/genes11010007)
Supplement: Supplementary file 1 [file genes-11-00007-s001.pdf]

Table S1 Reported metal resistance prokaryotes and their genome GC content

Species	Resistance	Genome accession number	Median GC%	Reference
Comamonas testosteroni S44	Zinc	ADVQ00000000	61.25	[3]
Comamonas testosteroni KF-1		AAUJ02000001	61.5	[4]
C. testosteroni CNB-2		CP001220	61.25	[5]
Mesorhizobium metallidurans	Cadmium	NZ_CAUM01000143	62.5	[6]
Gluconacetobacter diazotrophicus		NC_011365	66.3	[7]
Cupriavidus alkaliphilus (strains ASC-732, MLR2-44, MtRBr-3211, CAG-122)	Copper	GCA_900094595.1<br>GCA_003254285.1<br>GCA_003268995.1<br>GCA_003096375.1	67.6-67.9	NA
Cupriavidus basilensis		NZ_CP010536.1<br>NZ_CP010537.1	65.2111	[28]
Cupriavidus campinensis		CP043440.1	66.0901	NA
Cupriavidus gilardii		NZ_CP010516.1<br>NZ_CP010517.1	67.4<br>67.8	[29]
Cupriavidus metallidurans		NC_007973.1<br>NC_007974.2	63.8<br>63.6	[30]
Cupriavidus nantongensis		NZ_CP014844.1<br>NZ_CP014845.1	66.2<br>67.7	NA
Cupriavidus necator		NC_015726.1<br>NC_015723.1	66.5<br>66.7	[31]
Cupriavidus oxalaticus (strains X32, LMG 2235, NBRC 13593)		NZ_CP038634.1<br>NZ_CP038635.1<br>NZ_LT978515.1	66.7-66.9	NA
Cupriavidus pauculus		NZ_BBQN00000000.1	64.8	[32]
Cupriavidus pinatubonensis		NC_007347.1<br>NC_007348.1	64.7<br>65	NA
Cupriavidus alkaliphilus		NZ_FMAD00000000.1	67.8	NA
Cupriavidus plantarum (strains SLV-132, MA1-2za, MA1-4a)		GCA_003148725.1<br>GCA_003386635.1<br>GCA_003664285.1	65.9-66.0	NA
Cupriavidus taiwanensis (44 strains)		NC_010528.1<br>NC_010530.1<br>NZ_LT984801.1<br>NZ_LT984806.1	66.7-67.9	NA
Thiobacillus denitrificans (strains ATCC 25259, ASM153012v1, DSM 12475)	Heavy metals and/or acid	NC_007404.1<br>NZ_LDUG00000000.1<br>NZ_AQWL00000000.1	62.8	[33]
Thiobacillus sp. (24 strains)		GCA_001725525.1<br>GCA_001899305.1<br>GCA_001897705.1<br>GCA_008015195.1	62.74	[34]
Thiobacillus thioparus		NZ_ARDU00000000.1	62.3	NA
Rubrobacter aplysinae	Radiation and/or heavy metals	NZ_LEKH00000000.1	65.1	[35]
Rubrobacter indicoceani		NZ_CP031115.1	63.7	NA
Rubrobacter radiotolerans		NZ_CP007514.1	66.6	NA
Rubrobacter taiwanensis		NZ_SKBU00000000.1	67.2	NA
Rubrobacter xylanophilus		NC_008148.1	69.6	[36]
Deinococcus metallilatus (strains MA1002, MA1002-m5)	Radiation, desiccation and/or heavy metals	NZ_CP038512.1	68.8	NA
Deinococcus actinosclerus (strains BM2, SJTR)		NZ_CP013910.1<br>NZ_CP029774.1	70.6	NA
Deinococcus radiophilus		NZ_RXPE00000000.1	62.7	NA

Deinococcus irradiatisoli		67	NA
Deinococcus koreensis	NZ_PPPD00000000.1	69.5	NA
Deinococcus aerius	NZ_BFAG00000000.1	69.9	[37]
Deinococcus planocerae	NZ_PNOR00000000.1	70.2	NA
Deinococcus indicus	NZ_NHMK00000000.1	69.8	NA
Deinococcus reticulitermitis	NZ_FNZA00000000.1	68.6	NA
Deinococcus puniceus	NZ_CP011387.1	62.6	[38]
Deinococcus swuensis	NZ_CP010028.1	67.4	NA
Deinococcus phoenicis	NZ_JHAC00000000.1	69	[39]
Kineococcus radiotolerans	NC_009664.2	74.2	[40]
Deinococcus radiodurans (strains R1, R1, ATCC 13939, DSM 20539, UBA8872)	NC_001263.1<br>NC_001264.1<br>NZ_CP015081.1<br>NZ_CP015082.1<br>JHYL01<br>JMLF01<br>DPCQ01	66.6	[41-43]
Deinococcus grandis	NZ_BCMS00000000.1	69.9	[43]
Deinococcus peraridilitoris	NC_019793.1	63.7	NA
Deinococcus wulumuqiensis	NZ_AXLL00000000.1	66.3	[44]
Deinococcus frigens	NZ_JNIW00000000.1	65.4	NA
Deinococcus yavapaiensis	NZ_QJSX00000000.1	65.4	NA
Deinococcus pimensis	NZ_AZYF00000000.1	69.3	NA
Deinococcus aquatilis	NZ_ARKH00000000.1	62.1	NA
Deinococcus apachensis	NZ_ARLS00000000.1	68.7	NA
Deinococcus radiopugnans	NZ_VDMO00000000.1	67.1	[45]
Deinococcus ficus	NZ_AUMV00000000.1	67	NA
Deinococcus murrayi	NZ_AXWT00000000.1	69.4	NA
Deinococcus marmoris	NZ_JNIV00000000.1	64.3	NA
Deinococcus deserti	NC_012526.1	63	[44, 46]
Deinococcus maricopensis	NC_014958.1	69.8	[47]
Deinococcus gobiensis	NC_017790.1	69.2	[48]
Deinococcus proteolyticus	NC_015161.1	65.7	[49]
Deinococcus hopiensis	NZ_FWWU00000000.1	64.9	NA
Deinococcus geothermalis	NC_008025.1	65.6	[50]
